# Effectiveness of referral to a population-level telephone coaching service for improving health risk behaviours in people with a mental health condition: study protocol for a randomised controlled trial

**DOI:** 10.1186/s13063-021-05971-6

**Published:** 2022-01-17

**Authors:** Grace Hanly, Elizabeth Campbell, Kate Bartlem, Julia Dray, Caitlin Fehily, Tegan Bradley, Sonya Murray, Christophe Lecathelinais, John Wiggers, Luke Wolfenden, Kate Reid, Tahlia Reynolds, Jenny Bowman

**Affiliations:** 1 School of Psychological Sciences, University of Newcastle, Callaghan, NSW Australia; 2 Hunter Medical Research Institute, Clinical Research Centre, New Lambton Heights, Australia; 3 Priority Research Centre for Health Behaviour, School of Medicine and Public Health, University of Newcastle, Callaghan, NSW 2308 Australia; 4 School of Medicine and Public Health, University of Newcastle, Callaghan, NSW 2308 Australia; 5 Population Health, Hunter New England Local Health District, Wallsend, NSW Australia; 6 NSW Office of Preventive Health, Liverpool, NSW Australia

**Keywords:** Health behaviour, Mental health, Chronic disease, Exercise, Diet, Telephone service, Health coaching, Behaviour change, Randomised controlled trial

## Abstract

**Background:**

People with a mental health condition have a shorter life expectancy than the general population. This is largely attributable to higher rates of chronic disease and a higher prevalence of modifiable health risk behaviours including tobacco smoking, alcohol consumption, poor nutrition, and physical inactivity. Telephone support services offer a viable option to provide support to reduce these health risk behaviours at a population-level; however, whilst there is some research pertaining to Quitlines, there is limited other research investigating whether telephone services may offer effective support for people with a mental health condition. This protocol describes a randomised controlled trial that aims to evaluate the referral of people with a mental health condition to a population-level telephone coaching service to increase physical activity, healthy eating, or weight management, and increase attempts to do so.

**Methods:**

A parallel-group randomised controlled trial will be conducted recruiting participants with a mental health condition through community mental health services and advertisement on social media. Participants will be randomly assigned to receive either a health information pack only (control) or a health information pack and a proactive referral to a free, government-funded telephone coaching service, the NSW Get Healthy Coaching and Information Service® (intervention), which offers up to 13 telephone coaching calls with a University Qualified Health Coach to assist with client-identified goals relating to physical activity, healthy eating, weight management, or alcohol reduction. Data will be collected via telephone surveys at baseline and 6 months post-recruitment. Primary outcomes are as follows: (1) minutes of moderate to vigorous physical activity per week, (2) serves of fruit consumed per day, (3) serves of vegetables consumed per day, and (4) a composite measure assessing attempts to change at least one health risk behaviour (any attempts to change physical activity, fruit consumption, vegetable consumption, or other parts of nutrition). Secondary outcomes include weight and body mass index.

**Discussion:**

This study is the first to evaluate the effectiveness of referral to a population-level telephone support service for reducing health risk behaviours relating to physical activity, healthy eating, and weight in people with a mental health condition. Results will inform future policy and practice regarding the delivery of telephone-based behaviour change coaching services and the management of physical health for this population to reduce health inequity and the burden of chronic disease.

**Trial registration:**

The Australian New Zealand Clinical Trials Registry ACTRN12620000351910. Retrospectively registered on 12 March 2020

## Administrative information

**Table:** 

Title	Effectiveness of referral to a population-level telephone coaching service for improving health risk behaviours in people with a mental health condition: study protocol for a randomised controlled trial
Trial registration	ACTRN12620000351910 [Australian New Zealand Clinical Trials Registry (ANZCTR)] [http://www.anzctr.org.au/Default.aspx] [retrospectively registered on 12 March 2020]
Protocol version	Version 9 of 11/12/2020
Funding	This research is funded by Cancer Council NSW.
Author details	Grace Hanly - 1, 2, 3. Elizabeth Campbell - 2, 3, 4, 5. Kate Bartlem - 1, 2, 3. Julia Dray - 1, 2, 3. Caitlin Fehily – 1, 2, 3. Tegan Bradley - 1, 2, 3. Sonya Murray - 1, 2. Christophe Lecathelinais – 1, 2, 3. John Wiggers - 2, 3, 4, 5. Luke Wolfenden - 2, 3, 4, 5. Kate Reid - 6. Tahlia Reynolds - 6. Jenny Bowman - 1, 2, 3 1. School of Psychological Sciences, University of Newcastle, Callaghan, NSW, Australia. 2. Hunter Medical Research Institute, Clinical Research Centre, New Lambton Heights, Australia. 3. Priority Research Centre for Health Behaviour, School of Medicine and Public Health, University of Newcastle, Callaghan, NSW 2308, Australia. 4. School of Medicine and Public Health, University of Newcastle, Callaghan, NSW 2308, Australia. 5. Population Health, Hunter New England Local Health District, Wallsend, NSW, Australia. 6. NSW Office of Preventive Health, Liverpool, NSW, Australia.
Name and contact information for the trial sponsor	Investigator initiated trial; Jenny Bowman (Principal Investigator) jenny.bowman@newcastle.edu.au
Role of sponsor	This is an investigator-initiated trial. Therefore, the funders played no role in the design of the study and collection, analysis, and interpretation of data and in writing the manuscript.

## Background

Internationally, people with a mental health condition are estimated to have a life expectancy of up to 32 years shorter than the general population, with a median of 10 years life lost [1–3]. In Australia, this gap is estimated at up to 16 years [4]. This is mainly attributed to higher morbidity and mortality rates as the result of chronic diseases such as cardiovascular disease, type 2 diabetes, and cancer [1–3], as well as a higher prevalence of associated modifiable health risk behaviours including tobacco smoking [5–7], harmful alcohol consumption [8], poor nutrition [9], and inadequate physical activity [10]. These behaviours also substantially contribute to biomedical risk factors which are also more prevalent among people with a mental health condition, including high blood pressure and overweight and obesity [8], further increasing the risk of chronic diseases. The need to address these health risk behaviours in this population has been identified as a key initiative to help close the gap in health equity, both internationally by organisations including the World Health Organisation [11] and in Australia and national and state level [12, 13].

Systematic review evidence has demonstrated that telephone-delivered coaching is an effective approach for behaviour change support in the general population with respect to physical activity [14], nutrition [14], and smoking cessation [15]. For instance, a systematic review and meta-analysis examining 27 comparisons from 25 studies published between 2006 and 2010 (22 randomised controlled trials (RCTs) and 3 dissemination studies), found strong evidence that telephone-delivered interventions helped clients in the general population to improve nutrition (commonly measured as fruit and vegetable consumption or saturated fat intake), and increase physical activity (often measured in minutes of moderate to vigorous activity or kcal expenditure per week) [16]. Twenty of 27 comparisons demonstrated a positive outcome for initiating changes in risk behaviour, determined by end-of-intervention improvements for at least 50% of participants in their focus behaviour compared to controls such as usual care or information materials [16]. However, research examining the potential of telephone-delivered interventions to reduce health risk behaviour in people with a mental health condition specifically is very limited, and no systematic reviews could be identified. An updated Cochrane review of 104 studies investigating telephone counselling for smoking cessation found evidence of benefit for both proactive (smokers initiating contact with service) telephone counselling support, increasing the likelihood of cessation from 7 to 10% and reactive (service initiating contact with smokers) telephone counselling support from 11 to 14% [15]. The review also found that Quitlines offering multiple calls tended to achieve greater smoking cessation rates than those offering only one coaching call.

For behaviours other than smoking, published research investigating telephone-delivered interventions for people with a mental health condition to date is limited to small feasibility trials and secondary analyses of service data [17–19]. For example, a small randomised controlled trial assessed the feasibility of a telephone-delivered intervention to increase physical activity in people with a serious mental illness who were overweight or obese [17]. The telephone counselling intervention provided pedometers plus individualised goal setting, behavioural counselling, and social support via telephone, and found a significant increase in physical activity in the intervention group relative to control (*n* = 8 per group at follow-up). A randomised-controlled feasibility trial conducted in Australia evaluated the telephone delivery of a healthy lifestyle intervention delivered by peer workers for people with a mental health condition [18]. With a sample of 43 participants, the study was not powered to find statistically significant effect sizes yet did observe a non-significant trend for improvement in fruit and vegetable consumption. The study also found incidental significant improvements in physical activity, despite this not being a target of the intervention. The authors reported good rates of trial retention and participation in intervention sessions, and high participant satisfaction ratings attributed to the telephone delivery.

As telephone coaching has been shown to be a viable, cost effective option for delivering health behaviour change support, it can be implemented at a population-level to provide large-scale support services [20]. Participants only require access to a telephone, providing substantial reach and overcoming many access and equity issues of face-to-face approaches. An extensive body of international research on population-level Quitlines has demonstrated their effectiveness in supporting smoking cessation; Quitline participants from the general population were 1.5 times more likely to have quit smoking 6 months after using the service than those using self-help materials [21]. In a number of countries, including Australia, research suggests that people with a mental health condition are well-represented among Quitline participants [22, 23]. For example, a study of 3132 Quitline registrants across three states in the US found almost half of survey respondents reported the diagnosis of at least one mental health diagnosis [24]; and in Victoria, Australia, 33.4% of 604 participants taking part in an evaluation of the state’s Quitline service 6 months after use disclosed a mental illness, including a primary diagnosis of: 52% depression, 22% anxiety, 13% schizophrenia, 10% bipolar disorder, and 3% other [23]. Data relating to the effectiveness of Quitlines for people with a mental health condition specifically is somewhat limited, but evidence from a number of countries does suggest this sub-group of smokers do benefit from Quitline participation although are significantly less likely to reach and sustain cessation [24, 25]. For example, quit rates for smokers 7 months after using a Quitline service is the USA were reported at 44% for those without a mental health condition compared to 37% for those with a mental health diagnosis [26]. Similarly in Australia, the evaluation of the Victorian Quitline in Australia found that participants with a mental health condition were just as likely to have made a quit attempt as those without; however, they were less likely to be successfully quit at both 1 month at 48% vs. 35% respectively and 6 months after using the service at 41% vs. 30% respectively [23].

Research investigating the effectiveness of population-level telephone coaching services for behaviours other than smoking, however, appears to be very limited. The only evaluation of such a service we could identify in the published literature relates to the NSW Get Healthy Coaching and Information Service® (GHS) in Australia: a free telephone coaching and support service based in NSW and available to the population of that state as well as South Australia and Queensland [27, 28]. A pre-post non-controlled evaluation of 1140 GHS participants who completed the service between February 2009 and December 2011, shortly after its introduction, found statistically significant reductions in biomedical risk factors including weight, waist circumference, and body mass index (BMI) [27]. Additional findings included a significant increase in physical activity, including the number of sessions of moderate and vigorous activity per week, and in nutrition, measured in serves of fruit and vegetables consumed per week. A recent study analysed service data from 11,925 GHS clients who enrolled in the coaching program between January 2015 and December 2017, which confirmed people with a mental health condition were making use of the GHS, with 26% of clients reporting a mental health condition [29]. A subsequent study of service data from 5629 GHS participants enrolled between January 2018 and October 2019, of whom 33% reported a mental health condition [30], compared service engagement and behavioural outcomes of clients with and without a mental health condition [30]. Those with a mental health condition were more likely to have actively withdrawn or been lost to follow-up and less likely to complete the coaching program (31% vs 36% completed). Among both participant groups, those who completed the program made significant improvements to fruit and vegetable consumption, time spent walking and moderate physical activity, weight, and BMI, whilst those without a mental health condition also made improvements to vigorous physical activity and waist circumference [30]. This evidence further supports the findings of the literature that suggests behaviour change may be more difficult for people with a mental health condition but shows they can improve health risk behaviours through use of a population-level telephone coaching service.

## Objectives

This study will assess the effectiveness of referral to the GHS to increase the following: (1) physical activity, (2) fruit, and (3) vegetable consumption, and (4) attempts to change these health risk behaviours for people with a mental health condition. It represents the first trial to date of the potential of such a population-level telephone coaching services for reducing these health risk behaviours for people with a mental health condition, and as such addresses not only a significant evidence gap, but the identified need to provide effective behaviour change support to this high-risk population.

## Methods/design

### Study design and setting

**Fig. 1:**
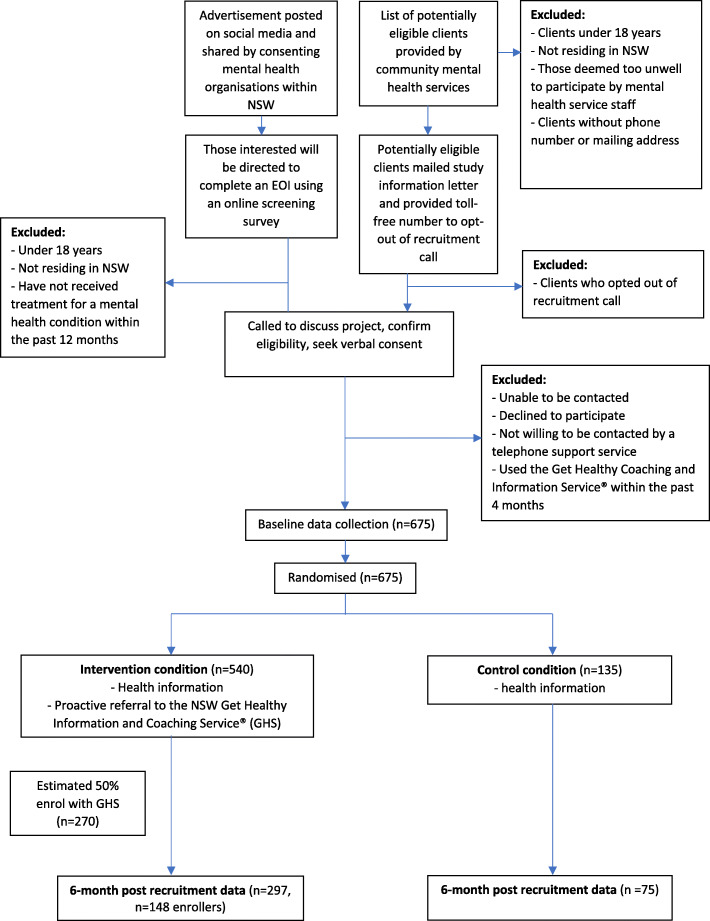
Study design flow diagram

A parallel-group RCT will be conducted and reported in accordance with Consolidated Standards of Reporting Trials (CONSORT) Statement [31]. The primary method of recruitment will include clients of community mental health services in two local health districts (Hunter New England and Central Coast) in New South Wales, Australia, which will be recruited to the study. The health districts encompass major city, regional, and rural populations. A second method of recruitment, added after the trial was registered, will recruit participants through study advertising on Facebook and the social media pages of mental health organisations within NSW (Fig. 1).

Participants will be randomly allocated to either the intervention condition (health information and proactive referral to a population-level telephone coaching service, the NSW Get Healthy Coaching and Information Service®) or the control condition (health information) in a 4:1 ratio respectively (Fig. 1). Target health behaviours and attempts to change these health behaviours will be assessed at baseline and 6 months post-recruitment using surveys conducted experienced Computer-Assisted Telephone Interview (CATI) staff. The intervention will be provided by the NSW Get Healthy Coaching and Information Service®(GHS), providing information and coaching for participants to set and achieve healthy lifestyle goals such as weight management, increasing physical activity, improving nutrition, and alcohol reduction [28, 32]. The service provided support to almost 14,000 coaching program clients and 1050 information only participants over a 2-year period from 2015 to 2017, with enrolment numbers increasing annually since the service began in 2009 [29].

This trial has been approved by the Hunter New England Human Research Ethics Committee (Ref no 2018/ETH00377) and the University of Newcastle Human Research Ethics Committee (approval No. H-2019-0013) and registered retrospectively with the Australian New Zealand Clinical Trials Registry (ACTRN12620000351910).

### Participants and recruitment

Clients will be eligible to participate if they (i) are 18 years or older, (ii) reside in NSW, (iii) have been diagnosed with a mental health condition and received mental health care from a community mental health service or other service provider in the past 12 months, (iv) have access to a telephone, and (v) are willing to be contacted by a telephone support service. Clients will be ineligible to participate if (i) they are deemed too unwell or clinically inappropriate to participate by staff of the community mental health services (for those recruited from community mental health services, described below), (ii) have no current phone number or mailing address, or (iii) they have used the GHS within the past 4 months.

To ensure target sample size is reached, several recruitment strategies will be utilised such as mail-outs to clients of community mental health services and expression of interest processes described below:

#### Community mental health services

Nine participating community mental health services across two local health districts in NSW will be asked to participate.

#### Clients

Participating mental health services will provide a list of all potentially eligible clients based on eligibility criteria i. and ii. and exclusion criteria i. and ii. Staff at the mental health service will review client lists and remove details of clients whom they identify as being too unwell or clinically inappropriate to participate (such as those in states of acute distress or some clients experiencing eating disorders). The list of potentially eligible clients will then be provided to the research team. Those without a listed mailing address or phone number will be removed. Eligible clients will be mailed a letter about the study by their mental health service, together with a detailed study brochure and a visual aid showing standard serving sizes for fruit, vegetables, and alcoholic drinks to assist with completing the telephone surveys. The letter will provide a toll-free number for clients to call if they wish to ‘opt out’ from being contacted further about the study. Approximately 2 weeks later, clients who do not opt out will receive an SMS advising that they will be called about the study within the next few days. Experienced telephone interviewers employed by one of the local health districts will then attempt to contact those clients to discuss the trial and confirm eligibility criteria. If participants are not available at that time, the interviewers will offer to arrange a convenient time to call back. Study posters and brochures will be placed in the service waiting rooms to provide context for the client letters. Mental health service staff will be advised of the study and asked to direct any client questions about the study to the project team.

#### Expression of interest

Advertisements for the study will be placed on social media pages of mental health organisations who provide services or advocacy groups within NSW. In addition, paid advertising through Facebook and Twitter will be utilised to promote the study, adjusting frequency and duration to promote participant recruitment. The advertisements will direct respondents to complete a brief online expression of interest (EOI) questionnaire to confirm eligibility criteria and collect contact information. Those persons deemed to be ineligible based on the information provided in the EOI form will see a response advising them of their ineligibility and will not be contacted by the interview team. A digital copy of the study brochure will be available to download from the EOI questionnaire, and the offer to receive a physical copy via mail will be included. The telephone interviewers will then contact such persons to provide further information about the study and confirm eligibility.

#### Consent

For participants who are eligible and wish to participate, telephone interviewers will confirm verbal consent to participate and complete the baseline survey. All telephone interviewers are trained and experienced in completing CATIs with populations of people with mental health issues. If at any time during the telephone call or baseline interview the interviewer feels the participant becomes distressed or is too unwell to participate, they will cease data collection and follow a protocol to raise any concerns for the safety and wellbeing of participants with the research team, who will then communicate with the mental health service to ensure adequate support is provided.

### Randomisation

A computer-generated random allocation sequence will be created by a statistician independent of the project to randomly allocate participants in a 4:1 ratio to the intervention and control groups respectively. The allocation sequence will use permuted-block randomisation with block sizes of 5, 10, and 15. A separate permuted-block schedule will be developed for each community mental health service and for those recruited through advertising. The allocation ratio will be used to account for participants that do not enrol in the GHS. The statistician will upload the random allocation sequence into REDCap (Research Electronic Data Capture) [33, 34]. The software embeds the sequence for use in the randomisation process; however, this is unable to be viewed or downloaded and therefore ensures concealment of allocation to the researchers until participants are assigned to treatment groups (control or intervention). At the end of the baseline survey, CATI interviewers will click the randomisation button in REDCap. Instructions will appear on the screen for CATI interviewers to ensure the participant is not informed of their group allocation.

### Blinding

Described above, the REDCap randomisation sequence ensures researchers and data collectors remain blinded to the allocation sequence until the baseline survey is complete [29, 30]. Whilst telephone interviewers will not directly state the allocation to the participant, they will inform intervention participants that they will be referred to the GHS. Due to the nature of the intervention, it is not possible for participants to be blinded to their allocation thereafter. When completing the follow-up data collection, telephone interviewers will not be informed of participants’ group allocation until the final section of the survey which includes questions for intervention participants about uptake of the GHS. Blinding before this point cannot be assured as participants may disclose their allocation by mentioning their experience with the service.

### Intervention

#### Control condition

Control participants will be mailed a health information pack including information on four common behavioural risks including smoking, nutrition, alcohol consumption, and physical activity, known as SNAP risk behaviours. These include written information and infographics about standard serving sizes; the health, social, and financial benefits of improving health behaviours; provide tips for making healthy changes; and relevant government websites focusing on healthy fruit and vegetable consumption.

#### Intervention condition

Intervention participants will be sent the same health information pack as the control group and the research team will provide the contact details to the GHS (proactive referral) using the GHS online referral form. In line with standard practice of the GHS, participants will then be contacted by the GHS by telephone to discuss the programs offered and determine if the client would like to enrol. Participants can choose to enrol in the coaching program or the Brief Intervention program, although only approximately 7% of clients chose this option [29]. Participants agreeing to either of these options will be considered to have enrolled with the GHS.

#### Coaching program

Participants will receive a copy of the GHS Program Information Booklet, which consists of an evidence-based information package about healthy eating, physical activity, and weight management in accordance with the Australian Guide to Healthy Eating [35] and the National Physical Activity Guidelines [36], and a journey booklet, which includes tracking tools and support tips to utilise throughout their coaching program. Coaching participants will complete an initial registration call to discuss their current eating and activity behaviours, determine their readiness to make changes, and set their personalised healthy lifestyle goals. They will then be allocated to a university-qualified health coach (such as a dietician, physiotherapist, or exercise physiologist) based on their identified goals. The service provides evidence-based support using motivational interviewing and self-regulation principles to develop motivation, overcome barriers, and sustain lifestyle changes including healthy eating, physical activity, healthy weight management, and alcohol reduction [32]. The coaching program consists of up to 13 proactive, individually tailored coaching calls with their health coach over six months to assist participants in setting healthy lifestyle goals and to provide educational material and support to achieve and maintain their lifestyle changes [32]. Specialised versions of the program exist for participants who are pregnant, identify as Aboriginal and/or Torres Strait Islander origin, are identified as being at risk of type-II diabetes, or have a primary goal of reducing alcohol consumption. Calls are tapered throughout the program, with more frequent calls during the first 12 weeks to assist with initiating behaviour change and less frequent calls during the second half of the program to support change maintenance [32]. Participants can stop the coaching program at any time, either by withdrawal if they have not achieved their health goal or as an ‘early graduation’ if they achieve their health goal in less than 13 calls and choose not to complete the remaining coaching calls. If participants wish to withdraw from the trial, they may advise their health coach, who will contact the trial management team, or contact the trial management team directly.

#### Brief intervention

Participants will receive the GHS Program Information Booklet by email or mail and are offered a one-off coaching call with a health coach for information and advice on these topics to supplement the written material [32]. These participants will also have the option to enrol in the 6-month coaching program at any point.

### Data collection procedures and measures

The primary outcomes of the trial include behaviour change outcomes: (1) minutes of moderate to vigorous physical activity conducted per week, (2) average serves of fruit consumed per day over the past month, (3) average serves of vegetables consumed per day over the past month, as well as a composite dichotomous outcome of (4) whether participants attempted to change any of the following health behaviours or biomedical risk factors over the past six months: increase physical activity, fruit consumption, vegetable consumption, any other dietary changes, weight, or alcohol consumption. Secondary outcomes for biometric risk factor change will include (1) self-reported weight (kg) and (2) body mass index (BMI). Secondary outcomes will also include dichotomous outcomes of attempts to change behaviours or biomedical risk factors individual, including (1) physical activity, (2) fruit consumption, (3) vegetable consumption, (4) other parts of their diet, (5) weight, or (6) alcohol consumption in the last 6 months.

**Fig. 2:**
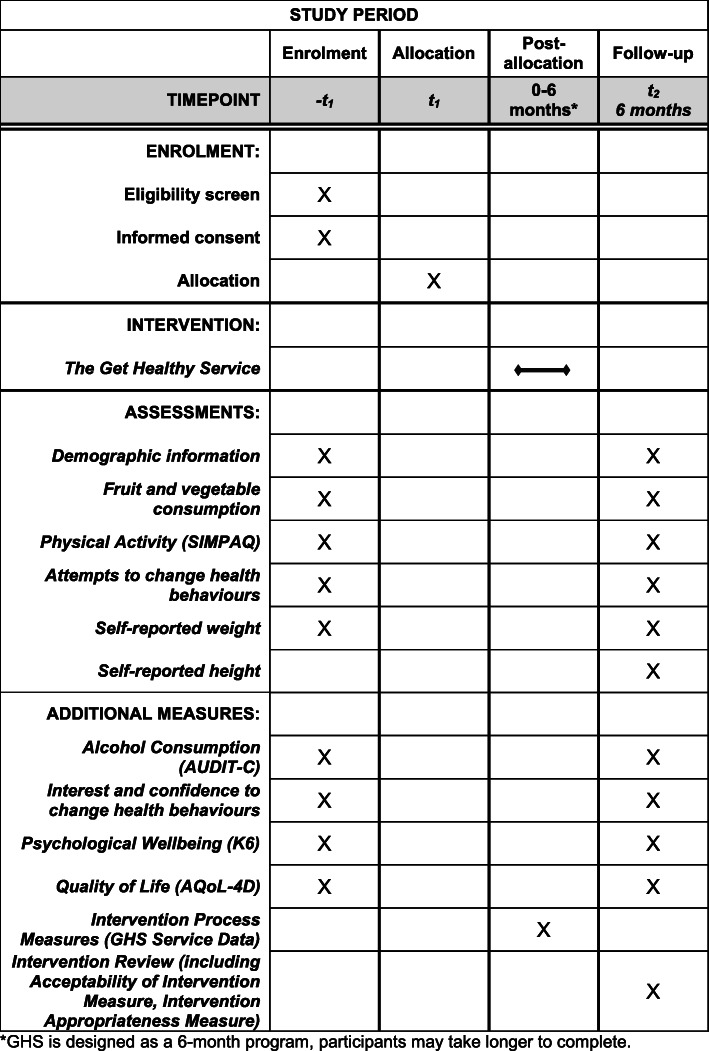
SPIRIT figure schedule of enrolment, intervention, and assessments

Data to assess primary and secondary outcomes, plus some additional variables listed below (see also Fig. 2), will be collected using CATI surveys by telephone interviewers experienced with working with mental health clients. The surveys will take approximately 30 min. During the interview, participants will be asked if they have the visual aid for serving sizes available. Those with the visual aid will be asked to refer to it throughout the survey; however, verbal explanations of these serving sizes based on Australian guidelines [35, 37] will be provided to all participants. Follow-up surveys will be completed using the same method approximately six months post-recruitment.

Data will also be obtained from the intervention service provider in the form of monthly reports on relevant contact variables for intervention group participants (see process outcomes below). The trial management team will attempt to contact any intervention participants who cease the intervention by passive withdrawal (do not graduate or terminate coaching but are unable to be contacted for a routine coaching call). Two attempts to contact the participant by telephone will be made. If the participant is successfully contacted, the trial management team will request permission to re-refer the participant to the GHS, who will reattempt to contact the participant and recommence the coaching program.

### Primary and secondary outcomes

The outcome and additional variables included in the survey will be as follows (at both baseline and follow-up unless stated otherwise):

#### Physical activity

The Simple Physical Activity Questionnaire (SIMPAQ) will be used to measure minutes of moderate to vigorous physical activity conducted per week. The SIMPAQ is a five-item self-report measure of physical activity over the past seven days which includes reported time spent in bed, sedentary and napping time, minutes of walking, minutes of physical activity, and minutes of other incidental activity [38]. Moderate-vigorous physical activity (MVPA) is calculated using the self-reported time in physical activity, time spent walking, and time spent exercising per week. The measure has been validated for people with a mental health condition [38]. The alternative SIMPAQ method for calculating sedentary time will be used, outlined by Rosenbaum et al., subtracting the total reported time spent on all activities from 24 h and confirming the remaining total with the participant as an accurate reflection of sedentary time per day [38].

#### Fruit and vegetable consumption

Participants will be asked to respond to single-item questions to identify the number of serves of fruit and vegetables consumed daily: e.g. “over the past month… how many serves of fruit do you eat in a usual day?” (number of serves/do not know). These items have been used in previous studies with a mental health population [39]. Participants with the visual aid available will be prompted to refer to the image guides for standard serving sizes of fruit and vegetables.

#### Attempts to change health behaviours

Six items will be used to record self-reported attempts to reduce health behaviours over the past 4 months, including “In the last four months, have you attempted to…”: increase physical activity, fruit consumption, or vegetable consumption; make any other changes to diet or nutrition; lose weight; reduce alcohol consumption (for participants that reported consuming any alcohol) (yes/no/do not know).

#### Biometrics

Height (cm or feet/inches) will be collected at baseline and self-reported weight (kg, pounds, or stone) in both baseline and follow-up surveys. These will be used to calculate a BMI score (kg/m^2^) [40].

### Additional measures

#### Alcohol consumption

Alcohol consumption will be measured using the short form of the Alcohol Use Disorders Identification Test (AUDIT-C) [41, 42]. Alcohol consumption is not a primary outcome as it is not the main focus of the GHS for most participants, with less than 1% of clients selecting an alcohol-reduction goal [29]; however, it has been included to measure any possible effects to other health risk behaviours addressed.

#### Participant characteristics

*Sociodemographic*: demographic information collected at baseline will include age, gender identity, highest level of education, current employment status, current marital status, and Aboriginal and/or Torres Strait Islander status. *Clinical*: primary and any secondary mental health diagnoses for which the participant is currently receiving or has received treatment for within the past 12 months and any physical health conditions that have required treatment within the past 2 months. These questions will be included in the baseline survey only. Current medications for mental health conditions will be recorded during baseline and follow-up surveys. *Health behaviour risk status*: calculated as per the Australian Guidelines for each health behaviour, with risk defined as follows; tobacco smoking, e.g. “how often did you smoke tobacco products in the last month?” (any use of tobacco products), and from the measures outlined above for alcohol consumption (AUDIT-C) [41, 42], physical activity (SIMPAQ) (< 150 min/week of MVPA) [36], and fruit (< 2 serves daily) and vegetable consumption (< 5 serves) [35].

#### Interest and confidence to change health behaviours

Participants will be asked to rate (i) their interest in making changes and (ii) their confidence in making changes to each of the six health behaviours. The ratings will be made on a Likert-type scale (1 to 10) where one represents ‘not at all interested/confident’ and 10 represents ‘extremely interested/confident’.

#### Psychological wellbeing

The six-item Kessler-6 (K6) [43] measure will be used to measure psychological wellbeing.

#### Quality of life

The 12-item Australian Quality of Life (AQoL-4D) [44, 45] has been validated for an Australian mental health population and will be used to measure quality of life.

#### Process measures

Process outcomes data provided by the GHS will include: whether contact has been made with the participant, enrolment in GHS, program selection (brief intervention, coaching), number of coaching calls, total call duration, and program completion status. Intervention participants will be advised that process data will also be provided by the GHS. Intervention participants will also be asked at follow-up about their experience with the service. Those who enrolled will be asked about program satisfaction, coaching and service delivery. Acceptability and appropriateness of the intervention will be assessed using the short 4-item versions of the Acceptability of intervention Measure and the Intervention Appropriateness Measure [46].

### Data management

All client data shared with the trial management team and GHS will be shared via secure data transfer link. No clinical information will be provided by the mental health services. Contact details will only be used to contact mental health service clients and potential participants about the study and confirm interest in participating. Details of clients who opt-out of contact will be removed. All participants are allocated a unique identification number and will be advised as part of the verbal consent process that their contact details will be released only to the trial management team and the GHS for intervention participants. All data will be stored in a REDCap database on a secure server held on site at the University of Newcastle and will only be accessed by ethics-approved researchers on the project and statisticians assisting with data analyses.

#### Auditing

Due to the nature of the intervention, the GHS will audit the intervention conduct as per their internal service procedures. Process data supplied by the GHS will also be used by researchers to monitor intervention conduct.

### Statistical analysis

Descriptive statistics will be used to describe baseline participant characteristics by group. Analyses will follow intention to treat principles, where participants are analysed according to their randomised treatment allocation. The initial analyses will be undertaken using all participants who provided baseline data. The three primary outcomes that are continuous variables (minutes physical activity, fruit serves, vegetable serves) will be analysed using linear mixed models, including a random intercept and random time effect for participant, as well as fixed effects for treatment group, time, and group*time interaction, and possible confounding variables including age, gender identity, and mental health diagnosis. Similar analyses will be conducted for continuous secondary outcomes (weight, BMI).

The same analyses using logistic mixed models will be conducted for dichotomous primary and secondary outcomes (attempts to change health behaviour variables).

As it is expected that approximately 50% of intervention participants will not enrol with the GHS (Fig. 1), an exploratory ‘per protocol’ analysis will be undertaken based on only those intervention participants who enrolled with the GHS (enrollers analysis). There are no interim analyses planned.

#### Sample size and power

The study will aim to recruit 675 participants (540 intervention and 135 control following the 4:1 allocation ratio). As per the intention-to-treat analysis, 55% of participants are anticipated to provide follow-up data (*n* = 297 intervention, *n* = 75 control, total 372). This final sample will provide an 80% power to detect a true difference between groups at follow-up of 0.58 serves of vegetables per day (SD = 1.6) [47], 0.47 serves of fruit per day (SD = 1.3) [35], and 109 min of moderate/vigorous physical activity per week (SD = 300) [48].

#### Protocol amendments

All protocol amendments will be submitted for ethics approval, updated in the ANZCTR, and identified in the future publication of trial outcomes.

#### Trial status

Recruitment for this trial commenced in October 2019 and is expected to be completed by January 2021.

#### Dissemination, ancillary, and post-trial care

The results of this trial will be disclosed in international, peer-reviewed journals. Both positive and negative results will be reported. Participating mental health services and participants who requested to receive information on trial outcomes will receive a laymen summary of results. Following completion of the follow-up survey, participants in the control condition will be informed about the GHS and provided with instructions to self-refer should they wish to enrol in the service. Participation in the trial will have no direct impact on current or future mental health treatment participants may be receive.

## Discussion

This is the first RCT designed to evaluate the effectiveness of a population-level telephone support service to reduce health risk behaviours in people with a mental health condition. The knowledge gained from this trial will contribute to the evidence regarding whether a telephone-delivered healthy lifestyle coaching program targeted to the general population can provide effective support to people with a mental health condition. The outcomes will inform program delivery and policy to maximise opportunities to reduce the differential rates of health behaviours, address the increased risk of preventable chronic disease, and improve life expectancy and quality of life for this vulnerable population.

## Trial status

Recruitment for this trial began in October 2019 and will continue until January 2021. Publication of this protocol was delayed due to the impacts of COVID19 requiring the introduction of additional recruitment sites and methods identified above.

## Data Availability

Deidentified datasets used and/or analysed during the current study will be made available from the corresponding author upon reasonable request using a secure data transfer link.

## Abbreviations

RCT: Randomised controlled trial
GHS: The NSW Get Healthy Coaching and Information Service® GHS
BMI: Body mass index
MVPA: Moderate-vigorous physical activity
